# Vagal modulation of resting heart rate in rats: the role of stress, psychosocial factors, and physical exercise

**DOI:** 10.3389/fphys.2014.00118

**Published:** 2014-03-24

**Authors:** Luca Carnevali, Andrea Sgoifo

**Affiliations:** Stress Physiology Laboratory, Department of Neuroscience, University of ParmaParma, Italy

**Keywords:** autonomic nervous system, heart rate variability, arrhythmias, stress, anxiety, depression, exercise, rat

## Abstract

In humans, there are large individual differences in the levels of vagal modulation of resting heart rate (HR). High levels are a recognized index of cardiac health, whereas low levels are considered an important risk factor for cardiovascular morbidity and mortality. Several factors are thought to contribute significantly to this inter-individual variability. While regular physical exercise seems to induce an increase in resting vagal tone, chronic life stress, and psychosocial factors such as negative moods and personality traits appear associated with vagal withdrawal. Preclinical research has been attempting to clarify such relationships and to provide insights into the neurobiological mechanisms underlying vagal tone impairment/enhancement. This paper focuses on rat studies that have explored the effects of stress, psychosocial factors and physical exercise on vagal modulation of resting HR. Results are discussed with regard to: (i) individual differences in resting vagal tone, cardiac stress reactivity and arrhythmia vulnerability; (ii) elucidation of the neurobiological determinants of resting vagal tone.

## Introduction

The sinoatrial node has an intrinsic rate of spontaneous automaticity that sets the basic rhythm of the heartbeat (Jose and Collison, 1970). The dynamic balance between the sympathetic and parasympathetic (vagal) influences on sinoatrial node activity primarily determines the actual heart rate (HR) of a given physiological state. In healthy individuals, vagal modulation (or “tone”) prevails under resting conditions. The role of resting vagal tone in healthy cardiac function has been increasingly recognized over the past 20 years (Levy and Schwartz, 1994). This was predominantly due to the rise of research that applied heart rate variability (HRV) analysis as a window into cardiac autonomic control. Mounting evidence indicates that those individuals showing higher vagal tone than average at rest tend to be more resilient to stress, adapting well across a number of different situations (El-Sheikh et al., 2001; Kok and Fredrickson, 2010; Smeets et al., 2010; Souza et al., 2013). On this regard, the beneficial effects of regular physical exercise on cardiac health appear to be mediated by an increase in resting vagal outflow (Smith et al., 1989; Rosenwinkel et al., 2001; Rennie et al., 2003; Soares-Miranda et al., 2009; Fu and Levine, 2013). On the contrary, low levels of vagal modulation may not adequately counteract sympathetic stimulation, leaving the heart vulnerable to ventricular tachyarrhythmias and sudden cardiac death (Schwartz et al., 1988; Esler, 1992; Volders, 2010). Several factors, including chronic life stress, negative personality traits, anxiety and mood states have been associated with vagal withdrawal and sympathetic predominance (Sloan et al., 1994; Friedman and Thayer, 1998; Rozanski et al., 1999; Gorman and Sloan, 2000; Lucini et al., 2005). Moreover, depression of vagal modulation characterizes cardiovascular pathology (e.g., heart failure) (Sabbah et al., 2011) and strongly predicts cardiac mortality after myocardial infarction (La Rovere et al., 1998). Interventional therapies for restoring the autonomic balance in such psychological and cardiac conditions include pharmacological, biobehavioral, and exercise strategies (Bryniarski et al., 1997; Iellamo et al., 2000; Tsai et al., 2006; Blumenthal et al., 2007; Nolan et al., 2008). Traditionally, pharmacological approaches have mainly been directed at reducing cardiac sympathetic outflow, while overlooking the possibility of enhancing vagal tone. This was presumably due to a lack of a comprehensive understanding of the central neurobiological determinants of resting vagal tone. Preclinical research is now attempting to fill this knowledge gap.

This paper focuses on current and past HRV research in rats that can potentially increase our understanding of the neurobiological mechanisms underlying vagal tone impairment/enhancement. It describes (i) how resting vagal tone is regulated and measured, (ii) the relations among individual differences in resting vagal tone, cardiac stress reactivity, and arrhythmia vulnerability, and (iii) the role of stress, psychosocial factors and physical exercise on the neural determination of resting vagal tone.

## Origin, components, and measurements of resting vagal tone

**Figure 1:**
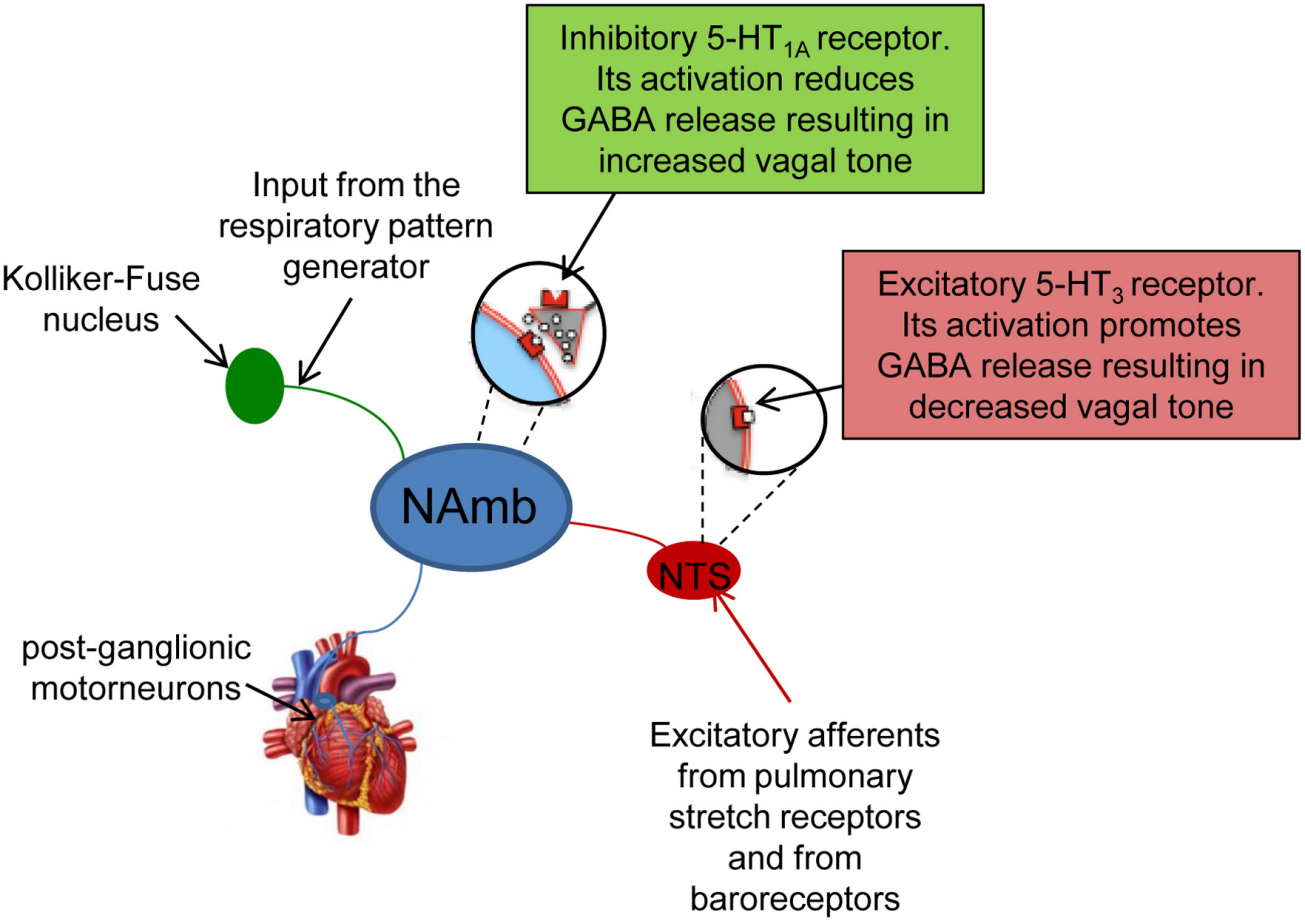
**Schematic representation of the origin and components of cardiac vagal pathway**. Vagal activity is generated by preganglionic vagal neurons in the nucleus ambiguus (NAmb), which are tonically active. They receive phasic respiratory-related inhibitory influences from the central respiratory pattern generator and from the nucleus tractii solitary (NTS). Their axons innervate post-ganglionic motoneurones located in a ganglionic plexus on the heart itself.

Cardiac vagal activity is generated by cardiac vagal pre-ganglionic neurones mainly located in the nucleus ambiguous (NAmb) of the lower brainstem (Figure 1). Their axons innervate post-ganglionic motoneurones located in a ganglionic plexus on the heart itself (Cheng et al., 1999; Cheng and Powley, 2000) (Figure 1). Under resting conditions, this vagal pathway fires a rapid and continuous signal (or “tonus”) to the sinoatrial node, slowing HR via acetylcholine release from efferent vagal nerve discharge. Vagally-released acetylcholine counteracts sympathetic effects both post-synaptically (via cAMP) and pre-synaptically (by reducing noradrenaline release from the sympathetic terminals) (Levy, 1971).

Cardiac vagal activity is dependent on excitatory and inhibitory synaptic inputs (Mendelowitz, 1996), including baroreflex [mediated by the nucleus tractus solitarii (NTS)] and respiratory input (Rentero et al., 2002) (Figure 1). Breathing modulates cardiac vagal activity through two mechanisms: (i) directly by projections from the central respiratory pattern generator, and (ii) indirectly by ascending afferents from lung stretch receptors that are activated during inspiration (Figure 1). These ascending afferents activate GABA-ergic neurons in the NTS that inhibit cardiac vagal motorneurons in the NAmb (Figure 1). Collectively, these mechanisms produce respiratory sinus arrhythmia (RSA)—rhythmic oscillations of HR around its mean value—with increases in HR during inspiration as vagal influence is momentarily suppressed, and decreases in HR during the early expiration phase as vagal influence resumes. Although moment-to-moment HR fluctuations can also be generated in response to, for example, physical movement and blood pressure and thermoregulatory changes, respiration is reliably periodic. Therefore, the strength of vagal influence can be estimated by measuring the rhythmic oscillation in the intervals between consecutive heart beats that are due to RSA—this is called HRV (Task Force, 1996).

HRV analysis is an established tool to estimate cardiac autonomic regulation in humans and animal models. Although the suitability of using HRV parameters to estimate sympathetic modulation is highly debated (Reyes Del Paso et al., 2013), this approach produces reliable measures of vagal tone (Berntson et al., 1997; Reyes Del Paso et al., 2013). Traditional HRV methods fall under the broader description of being either “time-domain” or “frequency domain” analyses. Among time domain measures of HRV, which are assessed with calculations based on statistical operations on R-R intervals (Kleiger et al., 1992), the root mean square of successive R-R interval differences (RMSSD) detects high frequency oscillations of HR, and therefore estimates parasympathetic nervous system activity (Stein et al., 1994). Frequency domain measures are based on spectral analysis of a sequence of R-R intervals and provide information on how power (variance) is distributed as a function of frequency. Usually, three oscillatory components are distinguished in the spectral profile: the very low (VLF), the low (LF), and the high (HF) frequency band. The power of HF band includes respiration-linked oscillations of HR and therefore reflects the modulation of vagus nerve discharge caused by respiration (Berntson et al., 1997), whereas the LF and VLF bands are related to a more gradual interplay between sympathetic and parasympathetic influences (Reyes Del Paso et al., 2013). The power of LF and HF bands is often reported in normalized (relative or fractional) units, which correspond to the relative value of each power in proportion to the total power (usually minus the VLF component). In particular, LF to HF ratio is taken as a synthetic measure of sympatho-vagal balance as it estimates the fractional distribution of power (Task Force, 1996).

It is worth mentioning for completeness sake that algorithms based on the chaos theory and nonlinear dynamics have been developed in order to evaluate in greater detail the intrinsic complexity of HRV (for an historical overview of the evolution of the concept of HRV and its application see Billman, 2011). Nonlinear methods are based on the assumption that the mechanisms involved in HR regulation interact with each other in a nonlinear way. The basic concept of nonlinear HRV methods is to try to capture the non-periodic behavior and complexity that exist inside the R-R interval dynamics. Various nonlinear methods have been tested in several sets of R-R interval data (Bigger et al., 1996; Lombardi et al., 1996; Makikallio et al., 1997, 1999; Huikuri et al., 1998, 2000), providing additional prognostic information and complementing traditional time- and frequency-domain analyses.

## Individual differences in resting vagal tone: physiological significance

Porges' polyvagal theory (Porges, 1995b) introduces a new perspective relating vagal tone during steady states (i.e., resting vagal tone) and vagal reactivity in response to environmental demands. According to this theory, measures of resting vagal tone are informative of an organism's ability to maintain homeostasis and the potential responsiveness of that organism. In particular, high levels of resting vagal tone can be considered a sign of autonomic flexibility, the capability of the parasympathetic nervous system to generate adequate responses to environmental challenges by modifying HR, respiration and arousal (Porges, 1995a,b, 2007; Beauchaine, 2001). On the contrary, depression of vagal modulation may predict mismatches between environmental demands and cardiac (re)-activity, thus increasing vulnerability to cardiac arrhythmias and sudden cardiac death (Schwartz et al., 1988; Esler, 1992; Volders, 2010).

**Figure 2:**
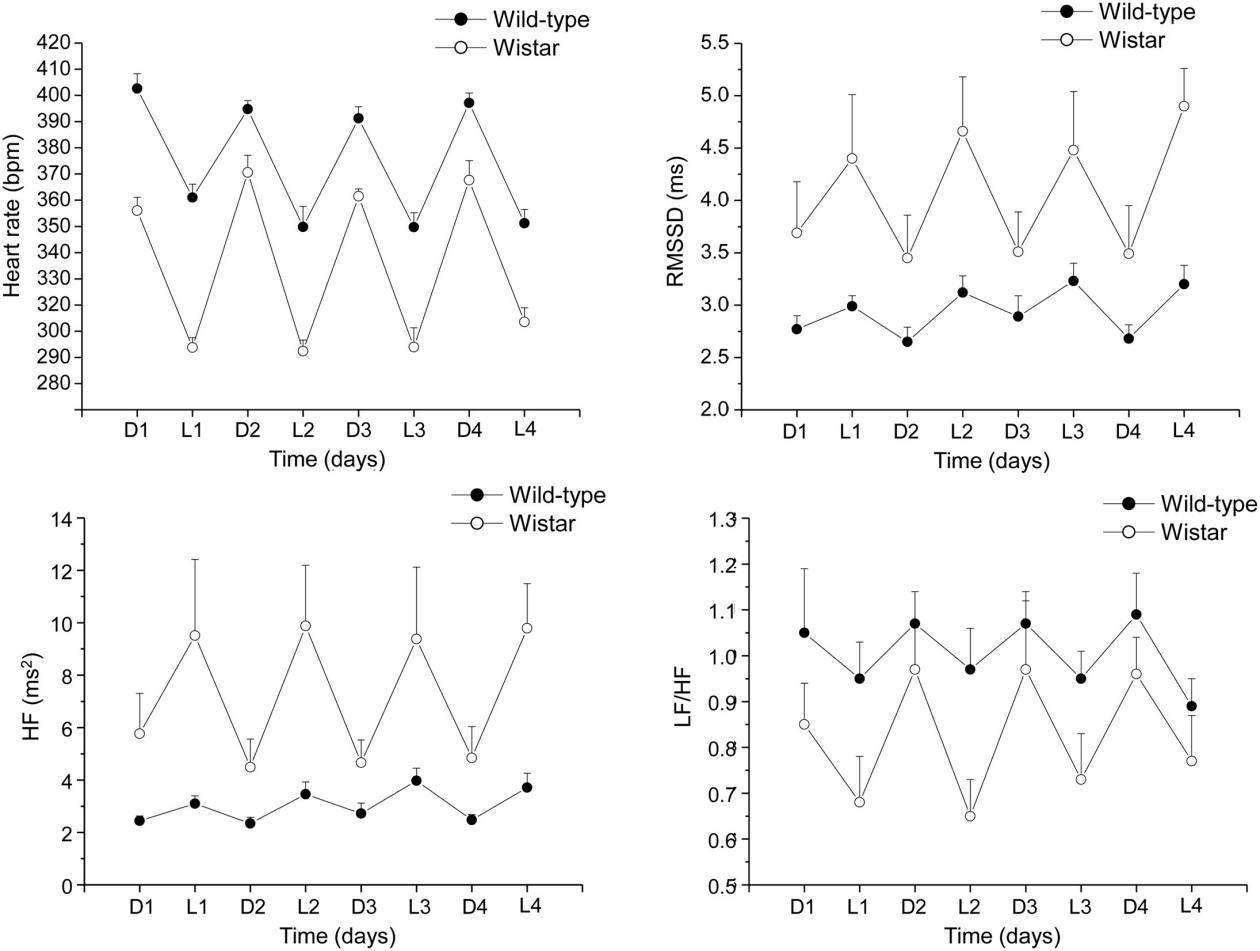
**Daily rhythms of heart rate, RMSSD, spectral power in HF band (0.75–2.5 Hz) and LF (0.2–0.75 Hz) to HF ratio in 4-month-old Wild-type (*n* = 10) and Wistar (*n* = 10) rats**. These values (reported as means ± s.e.m.) were obtained by averaging multiple 2-min segments acquired every hour during the 12 hours-light and 12 hours-dark phases, as previously described in detail (Carnevali et al., 2013a). Results of Two-Way ANOVA for repeated measures: group difference for heart rate (*F* = 103.7, *p* < 0.01), RMSSD (*F* = 6.8, *p* < 0.05) HF (*F* = 7.4, *p* < 0.5), and LF to HF ratio (*F* = 4.4, *p* < 0.05) values.

**Figure 3:**
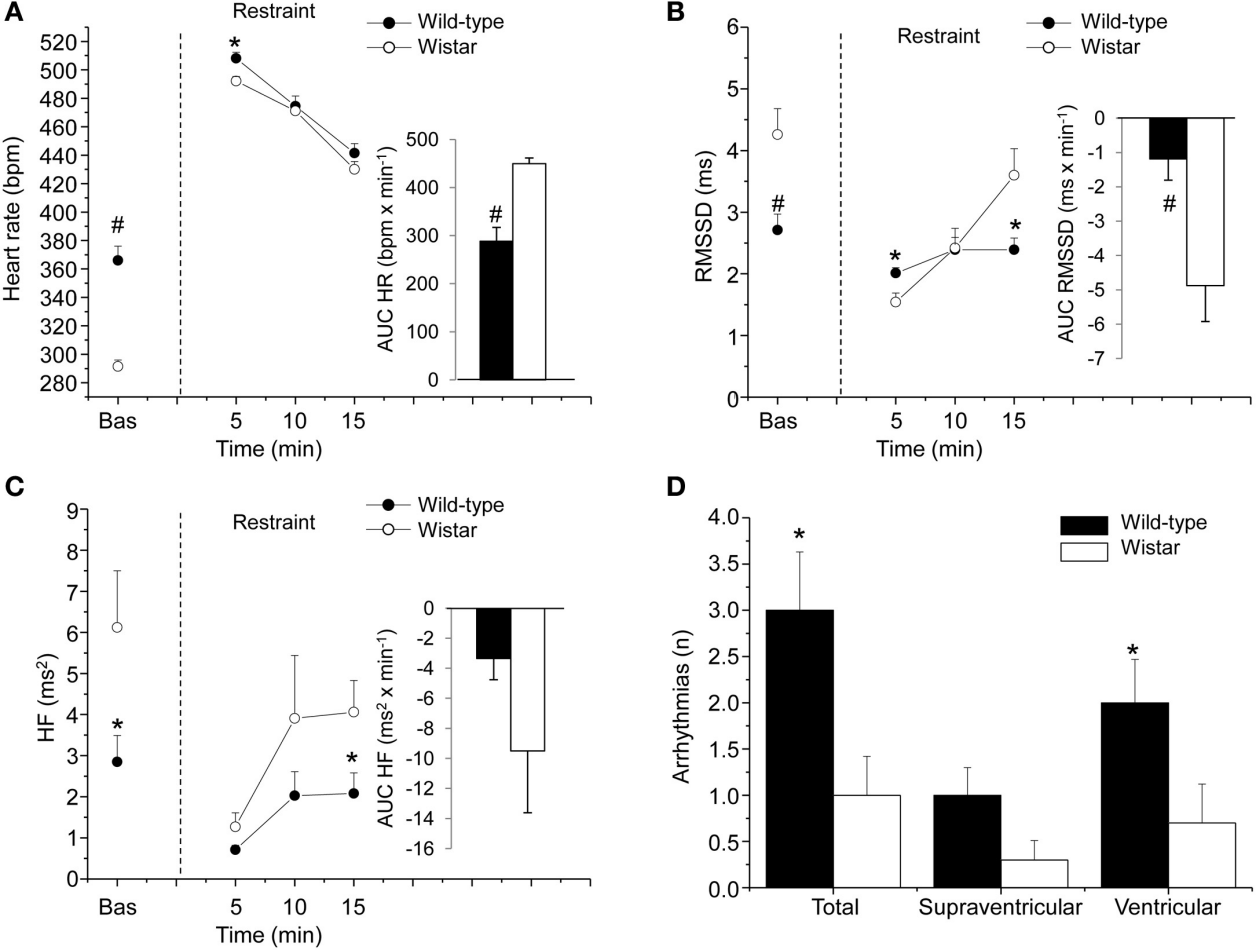
**Cardiac response to a restraint stress in 4-month-old Wild-type (*n* = 10) and Wistar (*n* = 10) rats**. Panels **(A–C)** show the time course of changes in heart rate, RMSSD values, and high-frequency (HF, 0.75–2.5 Hz) spectral power, respectively, in baseline conditions (bas, 30 min) and during the restraint test (15 min). Each point during the restraint phase is the mean of 5-min intervals. Inner graphs in **(A–C)** represent the area under the response time curve above baseline (AUC) of heart rate, RMSSD, and HF values during the restraint phase. Panel **(D)** illustrates the incidence of cardiac arrhythmias during the restraint phase. Data analysis was conducted as described previously in detail (Carnevali et al., 2013a). Values are reported as means ± s.e.m. ^*^ and ^#^ indicate a significant difference between HAB and LAB rats (*p* < 0.05 and *p* < 0.01, respectively; Student's *t*-test).

A deeper insight into the complex relations among individual differences in resting vagal tone, cardiac stress reactivity and arrhythmia vulnerability might be facilitated by HRV studies in rats. Conventional methods for HRV research in rats rely on conscious state electrocardiogram (ECG) recorded by telemetry (Sgoifo et al., 1996). By applying this approach, we obtained a detailed characterization of autonomic regulation of resting HR in two strains of rats, Wild-type and Wistar (Figure 2). Results indicate that Wild-type rats were characterized by higher HR than Wistar counterparts both during the dark/active and light/inactive phases of the daily cycle (Figure 2). HRV analysis allowed unveiling the autonomic determinants underlying the differences in resting HR between these two rat strains. As suggested by the vagal indexes RMSSD and HF power, Wild-type rats were clearly characterized by lower levels of vagal modulation compared to Wistar rats (Figure 2). Consequently, in Wild-type rats the sympatho-vagal balance (indexed by the LF to HF ratio) was shifted toward a sympathetic dominance (Figure 2). These results indicate that these two rat strains may be viewed as extremes, in terms of cardiac autonomic modulation, within the range of the natural variation of this species, and therefore may represent a valid model for investigating the neurobiological determinants underlying individual differences in resting vagal tone. As discussed before, this inter-individual variability may influence the individual's response to subsequent challenges. Addressing this issue, the two strains were tested under stress conditions (i.e., restraint test). Even though the peak HR reached in response to restraint stress was higher in Wild-type rats, these animals showed reduced overall HR stress responsiveness compared to Wistar rats, as suggested by the values of the area under the response curve above baseline (AUC) (Figure 3). In Wild-type rats, this phenomenon appeared to be related to a reduced vagal flexibility, namely a smaller vagal withdrawal in response to stress, and was coupled with a larger, although modest, vulnerability to arrhythmias compared to Wistar counterparts (Figure 3). Interestingly, a previous study demonstrated that arrhythmia vulnerability was remarkably larger in Wild-Type than Wistar rats in response to a social stressor (Sgoifo et al., 1998). Collectively, these findings support the view that low levels of vagal modulation of resting HR may be associated with an inability to flexibly generate adequate cardiac responses to stress, even in the absence of evident cardiovascular pathologies.

## Effects of stress and psychosocial factors on vagal modulation of resting HR

Several humans studies provide clear and convincing evidence that chronic life stress, negative personality traits such us aggressiveness, anger, and hostility, anxiety and mood states contribute significantly to the pathogenesis and progression of cardiac disorders (Sloan et al., 1994; Friedman and Thayer, 1998; Rozanski et al., 1999; Gorman and Sloan, 2000; Lucini et al., 2005; Albus, 2010). Putative underlying mechanisms may include a disruption of the sympatho-vagal balance, through an increase in sympathetic activity and/or a decrease in vagal tone. Rat studies on the integration of stress, psychosocial factors, and cardiac autonomic neural control have just started investigating the underlying mechanistic links.

### Stress

Several rat studies have investigated the short- and long-term effects of repeated stress exposure on cardiac autonomic regulation. In a study conducted by Trombini et al. (2012), rats submitted to intermittent episodes of restraint stress showed a prolonged increase in resting vagal drive in the days that followed the end of the stress period, as indicated by the increase in the vagal index RMSSD. Likewise, in another study, sub-chronically stressed rats (5 days of footsocks) exhibited a substantial increase in resting vagal tone that lasted well beyond the duration of the stressor (Carnevali et al., 2011). This peculiar phenomenon might be called “enduring vagal rebound” or “persistent vagal rebound” (Carnevali et al., 2011). In the literature, the term “vagal rebound” usually refers to a short-term vagal hyperactivity following a stressor, a sympathetic overdrive or reperfusion following acute myocardial infarction (Chiladakis et al., 2001; Mezzacappa et al., 2001). In this study (Carnevali et al., 2011), it was a relatively persistent, long-term consequence of repeated stress exposure, whose underlying neurobiological determinants have yet to be determined. However, it appears that this enduring vagal rebound is corticosterone- and serotonin-independent, as it was not prevented by metyrapone (inhibitor of corticosteroid synthesis) or fluoxetine (serotonin-selective reuptake inhibitor) treatments (Carnevali et al., 2011). Such sustained vagal activation after repeated stress exposure has been interpreted as a sign of adaptation, a transient compensatory phenomenon that initially overcomes the commonly observed stress-induced sympathetic hyperactivity (Carnevali et al., 2011). This adaptation might fail after a more prolonged exposure to stress and/or exposure to a more severe stressor, leading the organism to a maladaptive phase of vagal withdrawal and sympathetic dominance. Indeed, like humans, the most pervasive stressors in rats fall within the social domain (Bjorkqvist, 2001; Sgoifo et al., 2014). On this regard, the resident-intruder stress paradigm (Miczek, 1979) is regarded as an ethologically relevant model of psychosocial stress in rats, which involves subjecting a male intruder rat to aggressive threats and overt attacks by an unfamiliar male rat (resident) in the resident's home cage. This stress paradigm has recently been applied to study the shared pathophysiology that links stress-related psychological disorders such as anxiety and depression with cardiovascular disease (Carnevali et al., 2012b, 2013b; Wood et al., 2012; Sevoz-Couche et al., 2013). This raises another important issue that will be discussed in the next sections: the interplay between stress and related psychological disorders in the modulation of resting vagal tone.

### Anxiety

Cardiac autonomic function in rats displaying symptoms of stress-evoked anxiety was investigated in a study conducted by Sevoz-Couche et al. (2013). In this study, HRV analysis was conducted in rats submitted to intermittent daily episodes of social defeat, following a classical resident-intruder paradigm. Five days after the last defeat, stressed rats showed symptoms of an anxiety-like behavior that were accompanied by signs of reduced vagal modulation of resting HR, as suggested by the decrease in the vagal indexes RMSSD and HF power (Sevoz-Couche et al., 2013). Interestingly, such reduction in cardiac vagal tone was prevented by inhibition of the dorsomedial hypothalamus (DMH) with muscimol and the blockade of the NTS 5-HT_3_ receptors with granisetron. During acute stress, the DMH acts on the rostral cuneiform nucleus that, in turn, activates the rostral periacqueductal gray (Netzer et al., 2011). Downstream to this structure, serotonin (5-HT) is released to activate 5-HT_3_ receptors that are localized presynaptically on NTS vagal afferents (Leslie et al., 1990) (Figure 1). When activated, these receptors trigger glutamatergic activation of local GABAergic interneurons that project to the NAmb on pre-ganglionic vagal neurons, thereby inhibiting vagal activity (Loewy, 1990) (Figure 1). Therefore, it is reasonable to hypothesize that chronic activation of 5-HT_3_ receptors in the NTS due to overactivity of 5-HT in this region and/or hypersensitivity of 5-HT_3_ receptors is at the origin of vagal tone impairment in repeatedly stressed animals. These findings highlight the role of DMH and NTS in vagal tone impairment following repeated social challenge.

**Figure 4:**
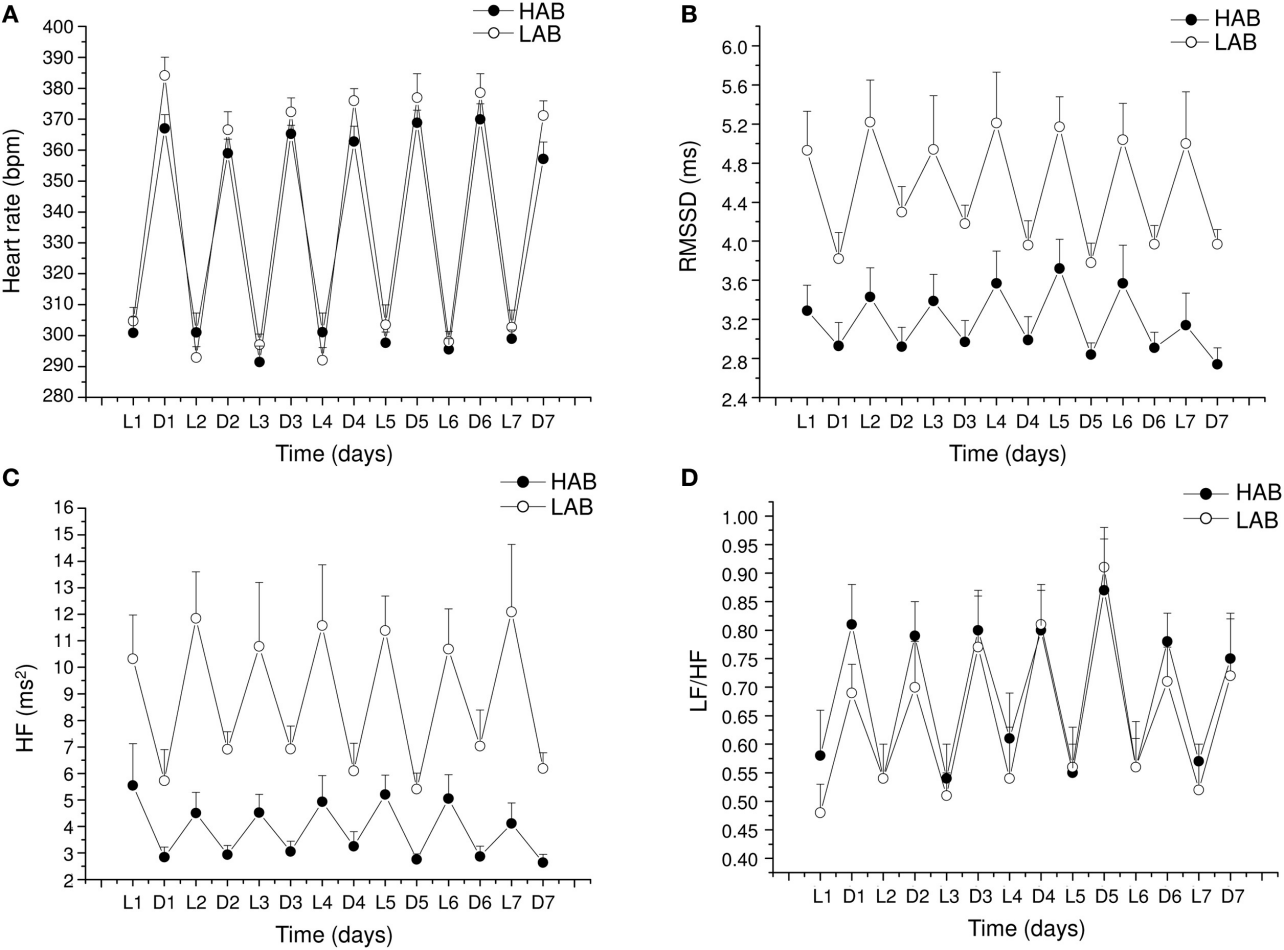
**Daily rhythms of (A) heart rate, (B) RMSSD, (C) spectral power in HF band (0.75–2.5 Hz) and (D) LF (0.2–0.75 Hz) to HF ratio in 4-month-old Wistar rats selectively bred for either high (HAB, *n* = 10) or low (LAB, *n* = 10) anxiety-related behavior**. These values (reported as means ± SEM) were obtained by averaging multiple 2-min segments acquired every hour during the 12 h-light and 12 h-dark phases, as previously described in detail (Carnevali et al., 2014). Results of two-way ANOVA for repeated measures: group difference for RMSSD (*F* = 16.4, *p* < 0.01) and HF (*F* = 17.48, *p* < 0.01) values. Modified from Carnevali et al. (2014).

**Figure 5:**
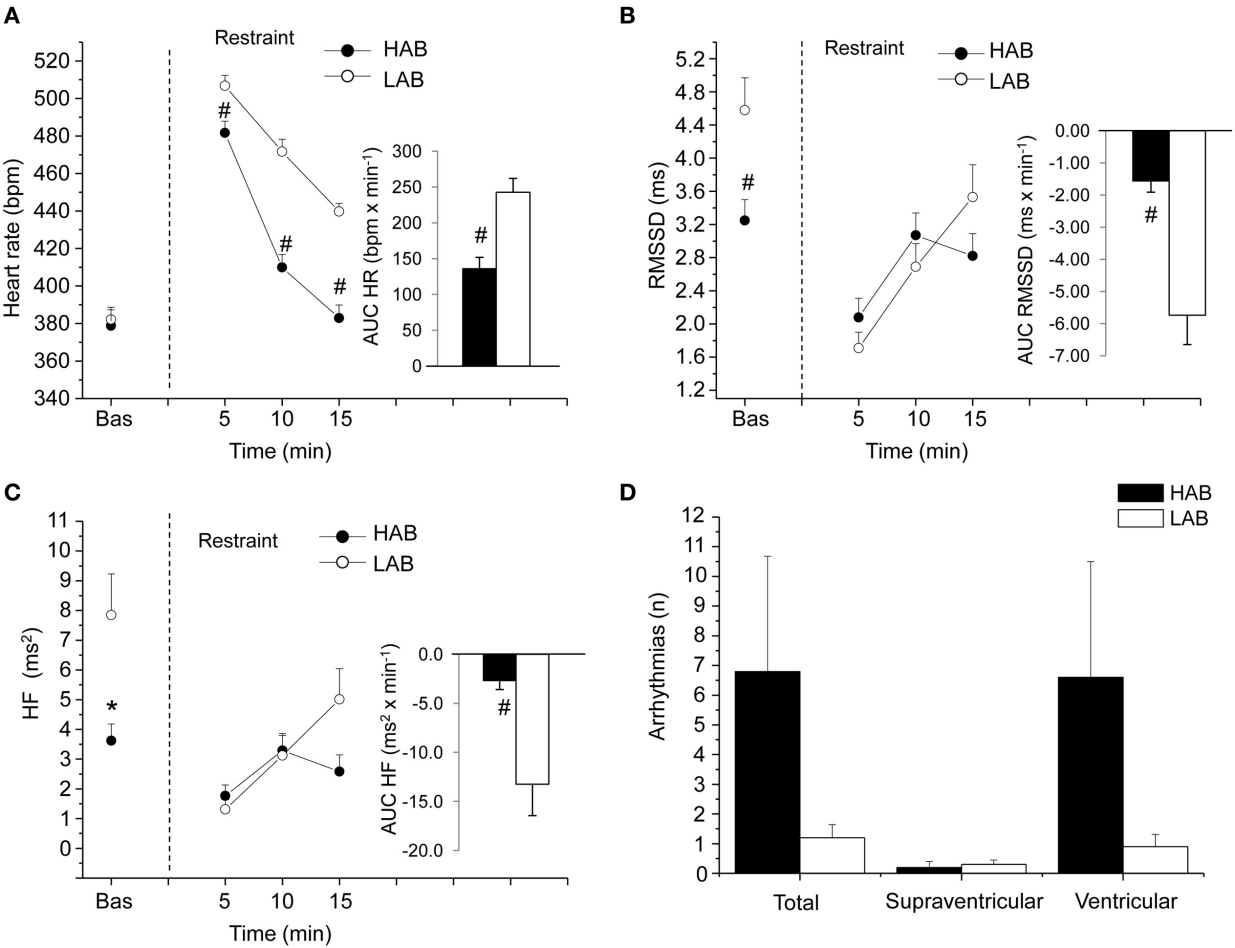
**Cardiac response to a restraint stress test in 4-mont-old Wistar rats selectively bred for either high (HAB, *n* = 10) or low (LAB, *n* = 10) anxiety-related behavior**. Panels **(A–C)** show the time course of changes in heart rate, RMSSD values and high-frequency (HF, 0.75–2.5 Hz) spectral power in baseline conditions (bas, 30 min) and during the restraint test (15 min), respectively. Each point during the restraint phase is the mean of 5-min intervals. Inner graphs in **(A–C)** represent the area under the response time curve above baseline (AUC) of heart rate, RMSSD and HF values during the restraint phase. Panel **(D)** illustrates the incidence of cardiac arrhythmias during the restraint phase. Values are reported as means ± s.e.m. ^*^ and ^#^ indicate a significant difference between HAB and LAB rats (*p* < 0.05 and *p* < 0.01, respectively; Student's *t*-test). Modified with permission from Carnevali et al. (2014).

The alterations in cardiac vagal tone that have been described in this animal model of stress-evoked anxiety may also characterize other forms of anxiety. Addressing this issue, we have recently investigated cardiac autonomic function in a rat model of trait anxiety, namely Wistar rats selectively bred for either high (HAB) or low (LAB) anxiety-related behavior (Carnevali et al., 2014). The HAB/LAB rats have been proved to display robust and consistent differences in their level of baseline anxiety (Landgraf and Wigger, 2002) and therefore represent a valid and reliable model for investigating the autonomic correlates of extremes in anxiety-related behavior. HAB rats clearly displayed a lower vagal modulation of resting HR (indexed by RMSSD and HF values) compared to LAB rats during both the light/inactive and dark/active phases of the daily cycle (Figure 4). One of the most puzzling findings was that, despite this evident difference in resting vagal tone, HAB and LAB rats had similar HR values (Figure 4). This is indicative of the fact that simple measurements of HR do not necessarily provide accurate insights into the functional regulatory characteristics of the autonomic nervous system. Indeed, mean HR values are indicative of the net effects of sympathetic and vagal influences on cardiac activity, which often result from a combination of concurrent changes in activity within both branches (Berntson et al., 1991). Therefore, it is reasonable to hypothesize that the reduced cardiac vagal tone observed in HAB rats was coupled with a decreased cardiac sympathetic influence on the sinoatrial node compared to LAB rats. Supporting this view, HAB and LAB rats showed similar LF to HF ratio (index of sympatho-vagal balance), suggesting that vagal and sympathetic influences on cardiac pacemaker activity were equally balanced in the two groups, leading to similar HR values (Figure 4). When tested under stress conditions (i.e., restraint test), HAB rats showed reduced HR stress responsiveness compared to LAB counterparts, as suggested by the values of the area under the response curve above baseline (AUC) (Figure 5). In HAB rats, this phenomenon appeared to be related to a reduced vagal flexibility, namely a smaller vagal withdrawal in response to stress, and was coupled with a tendentially larger vulnerability to ventricular arrhythmias compared to LAB counterparts (Figure 5). These findings support the view that a low tonic vagal modulation of HR in HAB rats may have determined an inability to flexibly generate adequate cardiac responses to environmental demands. Furthermore, these findings provide a strong basis for future mechanistic investigation aimed at defining, using this rat model, the central neural determinants of vagal control impairment in individuals with high levels of baseline anxiety.

### Depression

The complex interplay among social stress, depression and autonomic neural modulation of HR was investigated in a rat model of social stress by Wood et al. (2012). Rats exposed to 7 consecutive days of social defeat displayed symptoms of a depressive-like state. Forty eight hours after the final social defeat stress, depressed rats showed reduced HRV and signs of sympathetic predominance (increased LF to HF ratio). It was not determined whether such autonomic imbalance was due to a decrease in vagal tone and/or an increase in sympathetic tone. Similar changes in cardiac autonomic neural outflow were observed in rats submitted to a chronic mild stress model of depression (Grippo et al., 2004, 2006). Importantly, such abnormal modulation of resting HR in depressed rats was partially abolished by fluoxetine treatment (Grippo et al., 2006), suggesting a potential involvement of 5-HT neurotransmission in mediating autonomic changes in depressed individuals. Supporting this view, a study conducted by Hildreth et al. (2008) provided evidence of resting vagal tone impairment in a genetic rat model of depression (i.e., the Flinders-Sensitive Line rat) that was related to abnormal serotonergic control of vagal modulation of HR. Human studies indicate that brain 5-HT levels (Mann and Stoff, 1997) and 5-HT receptor function, particularly the 5-HT_1A_ receptors (Parsey et al., 2006), are abnormal in depression. Importantly, it has been demonstrated that chronic social defeat downregulates the 5-HT_1A_ receptor in the rat brain (Kieran et al., 2010). The 5-HT_1A_ receptors play an important role in inhibiting sympathetic neurons located in the medullary raphe area that activates the heart during stress (Nalivaiko, 2006; Nalivaiko and Sgoifo, 2009). In addition to sympathoinhibition, activation of 5-HT_1A_ receptors may have vagomimetic effects (Ngampramuan et al., 2008; Dutschmann et al., 2009; Carnevali et al., 2012a). Indeed, given that vagal preganglionic neurons in the NAmb are under tonic inhibition by GABA-ergic interneurons that express 5-HT_1A_ receptors, activation of such inhibitory 5-HT_1A_ receptors may lead to disinhibition of cardiomotor vagal neurons and consequently increase cardiac vagal tone (Jordan, 2005) (Figure 1). Therefore, a decreased density/function of 5-HT_1A_ receptors in these areas may be at the origin of the imbalance in the autonomic neural modulation of resting HR in chronically stressed and depressed individuals. Further work is required in order to determine whether serotonergic mechanisms do contribute to driving the changes in autonomic modulation of resting HR in rat models of depression.

### Aggressiveness

**Figure 6:**
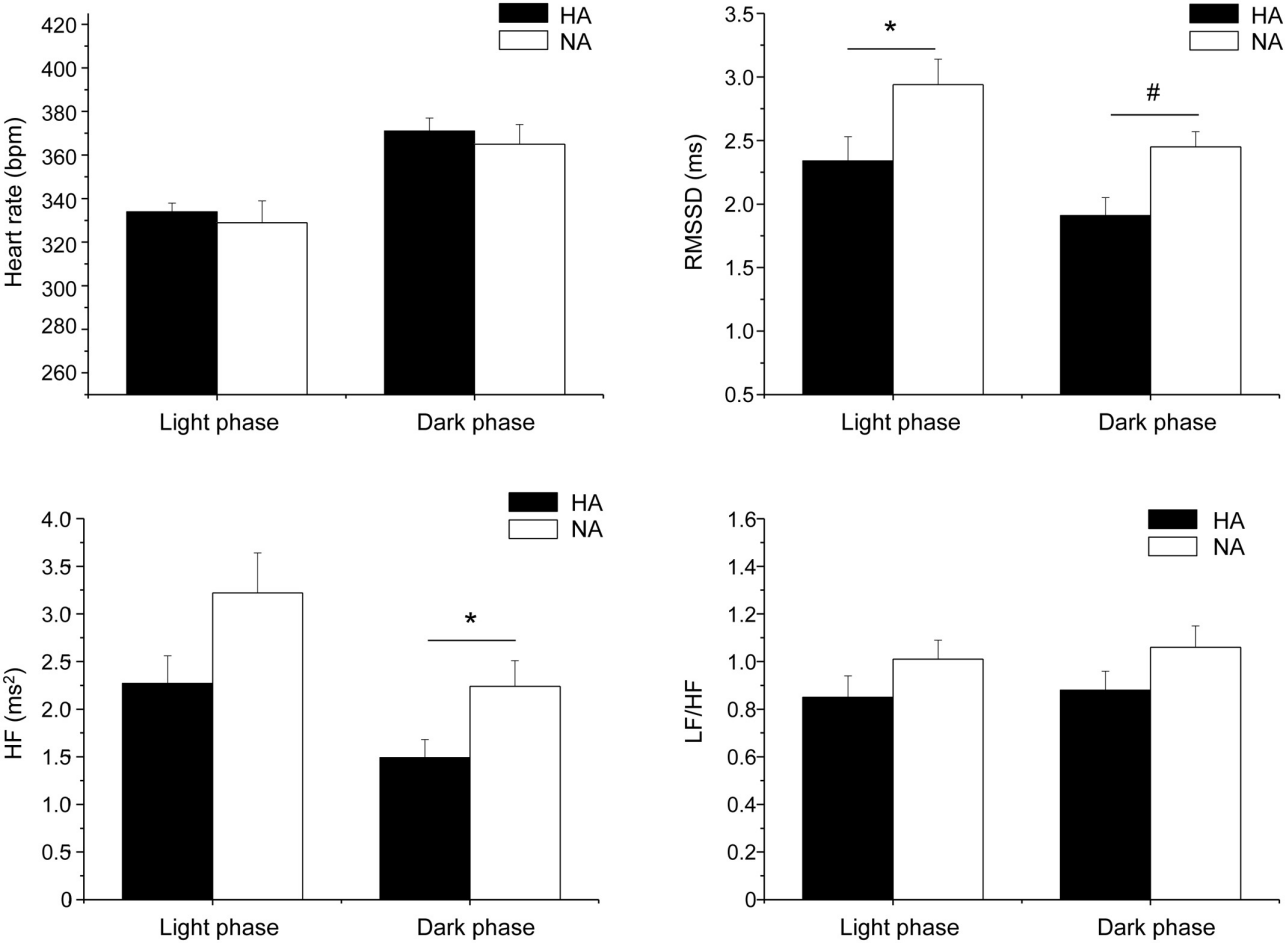
**Daily rhythms of heart rate, RMSSD, spectral power in HF band (0.75–2.5 Hz) and LF (0.2–0.75 Hz) to HF ratio in high-aggressive (HA, *n* = 10) and non-aggressive (NA, *n* = 10) Wild-type rats**. For the 12 hours-light and 12 hours-dark phases, values are reported as means ± s.e.m. of data obtained by averaging multiple 2-min segments acquired every hour over a period of 6 days. ^*^ and ^#^ indicate a significant difference between HAB and LAB rats (Student's *t*-test, *p* < 0.05 and *p* < 0.01, respectively). Modified with permission from Carnevali et al. (2013a).

**Figure 7:**
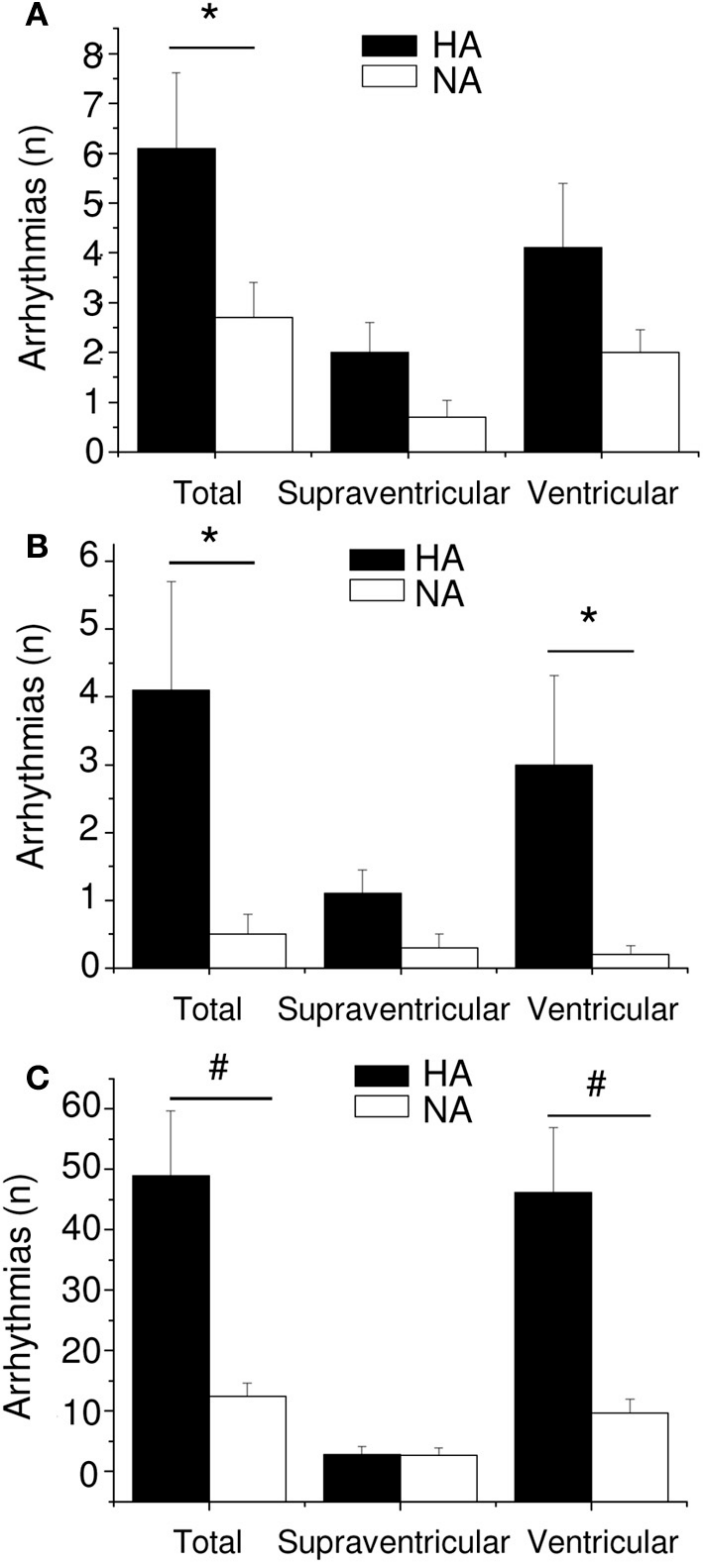
**Incidence of arrhythmias in high-aggressive (HA, *n* = 10) and non-aggressive (NA, *n* = 10) Wild-type rats during a restraint test (panel A), a psychosocial stress test (panel B) and following β-adrenoceptor pharmacological stimulation with isoproterenol (panel C)**. Values are reported as mean ± s.e.m. of number of events (n) per 15-min recording period. ^*^ and ^#^ indicate a significant difference between HA and NA rats (*p* < 0.05 and *p* < 0.01, respectively). Reproduced with permission from Carnevali et al. (2013a).

The association between personality traits (i.e., aggressiveness) and autonomic neural control of resting HR was the focus of one of our recent investigations (Carnevali et al., 2013a). In this study, high-aggressive (HA) and non-aggressive (NA) rats were selected from a population of adult male Wild-type rats on the basis of their latency time to attack a male intruder in a classical resident-intruder test performed on 3 consecutive days (HA: mean attack latency <90 s; NA: no overt aggression during the three tests, each lasting 600 s) (Koolhaas et al., 2013). As suggested by RMSSD and HF power indexes, HA rats exhibited lower levels of vagal modulation of resting HR during both the light/inactive and dark/active active phases of the daily cycle (Figure 6). However, despite different levels of resting vagal tone, HA and NA rats had similar HR values (Figure 6). Similarly to what has been argued above, it has been hypothesized that the reduced cardiac vagal tone observed in HA rats was coupled with a decreased sympathetic influence on sinoatrial node activity compared to NA rats. Consequently, vagal and sympathetic influences were equally balanced in the two groups (LF to HF ratio), leading to similar HR values (Figure 6) (Carnevali et al., 2013a). When tested under stress conditions (i.e., restraint and psychosocial stress test), HA and NA rats showed similar tachycardic responses. However, under these conditions HA rats displayed lower vagal antagonism and larger incidence of tachyarrhythmias compared to NA rats (Figure 7) (Carnevali et al., 2013a). In addition, injection of beta adrenergic agonist isoproterenol provoked a much larger incidence of ventricular tachyarrhythmias in HA rats compared to NA counterparts (Carnevali et al., 2013a) (Figure 7). The results of this study support the view that low levels of vagal modulation of resting HR are associated with arrhythmia susceptibility that may predict vulnerability to cardiac morbidity and mortality. Future mechanistic experiments using this rat model are needed in order to determine the neural determinants of vagal control impairment in aggressive individuals.

## Effects of physical exercise on vagal modulation of resting HR

Human studies provide evidence that regular physical exercise increases life expectancy in healthy individuals and reduces cardiac-related events in patients with coronary heart disease and heart failure (Rosenwinkel et al., 2001; Buch et al., 2002). The cardioprotective effects of physical training may be mediated, at least in part, by an increase in vagal modulation of resting HR (Crimi et al., 2009). However, central neural mechanisms that underlie these exercise-induced effects on vagal tone are not well understood. Cardiac autonomic adaptation induced by physical training has been subject of research in several carefully designed and rigorously conducted rat studies. These studies reported a clear decrease in resting HR in rats submitted to training with aerobic exercises (i.e., swimming, running) (Medeiros et al., 2004; Rossi et al., 2009; Souza et al., 2009; Sant'Ana et al., 2011; Neto et al., 2013). The effect of the autonomic nervous system in mediating training-induced resting bardycardia was investigated by means of (i) pharmacological approaches (after cardiac muscarinic and adrenergic blockade) and (ii) HRV analysis. Resting bradycardia after exercise training was mainly explained by a vagal effect (Medeiros et al., 2004) or by a reduction in intrinsic HR accompanied by a moderate increase in vagal modulation (indexed by HF spectral power values) (Rossi et al., 2009; Souza et al., 2009; Sant'Ana et al., 2011; Neto et al., 2013).

One possible limitation of these studies is that they applied protocols of forced exercise (treadmill or swimming). In doing so, the effects of physical exercise *per se* on cardiac autonomic function might have been confounded by the effects of accompanying psychological stress (the effects of repeated stress exposure on vagal modulation have been discussed before). On this regard, an elegant study conducted by Beig et al. (2011) investigated the effects of voluntary running on cardiac autonomic function. This study addressed another important issue: that is, whether the effects of voluntary exercise on autonomic function were long-lasting. This question is of major relevance, since loss of exercise-induced cardioprotection has been described soon after exercise cessation (Lin and Horvath, 1972). Rats submitted to a protocol of voluntary exercise exhibited resting bradycardia associated with increased vagal modulation (indexed by HF spectral power values) (Beig et al., 2011). Importantly, these effects persisted 10–12 days after termination of the training protocol, suggesting that voluntary exercise has an enduring effect on resting vagal tone.

Interestingly, voluntary training did not affect stress-induced tachycardia, but augmented resistance to cardiac arrhythmias, as evidenced by the higher doses of the proarrhythmic drug aconitine needed to provoke arrhythmic effects in trained rats (Beig et al., 2011). Such antiarrhythmic effect of exercise training seems to reflect electrophysiological changes (i.e., prolongation of the effective refractory period) induced by long-lasting increase in vagal outflow in trained rats (Beig et al., 2011). This suggests that the benefits of increased resting vagal tone induced by exercise training may be attributed to (i) an HR-lowering effect and (ii) other parasympathetic effects on the electrophysiological properties of the myocardium. However, the mechanistic links whereby physical training provokes changes in resting vagal outflow are currently unknown. One hypothesis is that exercise might reduce tonic GABA_A_ergic inhibition of neurons in the NTS involved in HR control (Figure 1), therefore increasing vagal influences on cardiac peacemaker activity (Mueller and Hasser, 2006). Given that physical exercise increases central 5-HT synthesis (Blomstrand et al., 1989), and that central 5-HT increases vagal modulation in conscious rats (Ngampramuan et al., 2008), it is tempting to speculate that activation of inhibitory 5-HT_1A_ receptors located on GABA-ergic interneurons in the Namb might mediate disinhibition of cardiomotor vagal neurons in that area (Figure 1). Further work is required in order to (i) evaluate potential correlations between duration/intensity of voluntary exercise and effects on resting vagal tone and (ii) provide a more comprehensive understanding of central neural mechanisms underlying exercise-induced vagal tone enhancement.

## Concluding remark

The influence of vagal tone on resting HR is highly variable among individuals. Several psychosocial and physiological conditions are thought to contribute significantly to this large inter-individual variability. From the data summarized in this paper, it is quite evident that rat studies can be extremely useful for investigating (i) the effects of stress, psychosocial factors, and physical exercise on vagal modulation of resting HR, and (ii) the consequences of such effects on cardiac stress reactivity and arrhythmia vulnerability. Moreover, the results of these studies have provided preliminary insights into the central neural mechanisms underlying vagal tone impairment/enhancement, which may be exploited by future experiments aimed at developing pharmacological approaches for enhancing vagal activity and cardioprotection.

### Conflict of interest statement

The authors declare that the research was conducted in the absence of any commercial or financial relationships that could be construed as a potential conflict of interest.

## Abbreviations

DMH: dorsomedial hypothalamus
HA: high-aggressive
HAB: high-anxiety behavior
HF: high frequency
HR: heart rate
HRV: heart rate variability
LAB: low-anxiety behavior
LF: low frequency
NA: non-aggressive
NAmb: nucleus ambiguus
NTS: nucleus tractus solitarii
RMSSD: root mean square of successive R-R interval differences
RSA: respiratory sinus arrhythmia
VLF: very low frequency
5-HT: serotonin.

## References

[B1] Albus C. (2010). Psychological and social factors in coronary heart disease. Ann. Med. 42, 487–494 10.3109/07853890.2010.515605 20839918

[B2] Beauchaine T. (2001). Vagal tone, development, and Gray's motivational theory: toward an integrated model of autonomic nervous system functioning in psychopathology. Dev. Psychopathol. 13, 183–214 10.1017/S0954579401002012 11393643

[B3] Beig M. I. Callister R. Saint D. A. Bondarenko E. Walker F. R. Day T. A. (2011). Voluntary exercise does not affect stress-induced tachycardia, but improves resistance to cardiac arrhythmias in rats. Clin. Exp. Pharmacol. Physiol. 38, 19–26 10.1111/j.1440-1681.2010.05456.x 21039755

[B4] Berntson G. G. Bigger J. T. Jr. Eckberg D. L. Grossman P. Kaufmann P. G. Malik M. (1997). Heart rate variability: origins, methods, and interpretive caveats. Psychophysiology 34, 623–648 10.1111/j.1469-8986.1997.tb02140.x 9401419

[B5] Berntson G. G. Cacioppo J. T. Quigley K. S. (1991). Autonomic determinism: the modes of autonomic control, the doctrine of autonomic space, and the laws of autonomic constraint. Psychol. Rev. 98, 459–487 10.1037/0033-295X.98.4.459 1660159

[B6] Bigger J. T. Jr. Steinman R. C. Rolnitzky L. M. Fleiss J. L. Albrecht P. Cohen R. J. (1996). Power law behavior of RR-interval variability in healthy middle-aged persons, patients with recent acute myocardial infarction, and patients with heart transplants. Circulation 93, 2142–2151 10.1161/01.CIR.93.12.2142 8925583

[B7] Billman G. E. (2011). Heart rate variability - a historical perspective. Front. Physiol. 2:86 10.3389/fphys.2011.00086 22144961 PMC3225923

[B8] Bjorkqvist K. (2001). Social defeat as a stressor in humans. Physiol. Behav. 73, 435–442 10.1016/S0031-9384(01)00490-5 11438372

[B9] Blomstrand E. Perrett D. Parry-Billings M. Newsholme E. A. (1989). Effect of sustained exercise on plasma amino acid concentrations and on 5-hydroxytryptamine metabolism in six different brain regions in the rat. Acta Physiol. Scand. 136, 473–481 10.1111/j.1748-1716.1989.tb08689.x 2473602

[B10] Blumenthal J. A. Sherwood A. Rogers S. D. Babyak M. A. Doraiswamy P. M. Watkins L. (2007). Understanding prognostic benefits of exercise and antidepressant therapy for persons with depression and heart disease: the UPBEAT study–rationale, design, and methodological issues. Clin. Trials 4, 548–559 10.1177/1740774507083388 17942470 PMC3677197

[B11] Bryniarski L. Kawecka-Jaszcz K. Bacior B. Grodecki J. Rajzer M. (1997). Effect of exercise rehabilitation on heart rate variability in hypertensives after myocardial infarction. J. Hypertens. 15, 1739–1743 10.1097/00004872-199715120-00082 9488232

[B12] Buch A. N. Coote J. H. Townend J. N. (2002). Mortality, cardiac vagal control and physical training–what's the link? Exp. Physiol. 87, 423–435 10.1111/j.1469-445X.2002.tb00055.x 12392106

[B13] Carnevali L. Bondarenko E. Sgoifo A. Walker F. R. Head G. A. Lukoshkova E. V. (2011). Metyrapone and fluoxetine suppress enduring behavioral but not cardiac effects of subchronic stress in rats. Am. J. Physiol. Regul. Integr. Comp. Physiol. 301, R1123–1131 10.1152/ajpregu.00273.2011 21795640

[B14] Carnevali L. Mastorci F. Audero E. Graiani G. Rossi S. Macchi E. (2012a). Stress-induced susceptibility to sudden cardiac death in mice with altered serotonin homeostasis. PLoS ONE 7:e41184 10.1371/journal.pone.0041184 22815962 PMC3399824

[B15] Carnevali L. Mastorci F. Graiani G. Razzoli M. Trombini M. Pico-Alfonso M. A. (2012b). Social defeat and isolation induce clear signs of a depression-like state, but modest cardiac alterations in wild-type rats. Physiol. Behav. 106, 142–150 10.1016/j.physbeh.2012.01.022 22330326

[B16] Carnevali L. Trombini M. Graiani G. Madeddu D. Quaini F. Landgraf R. (2014). Low vagally-mediated heart rate variability and increased susceptibility to ventricular arrhythmias in rats bred for high anxiety. Physiol. Behav. 128C, 16–25 10.1016/j.physbeh.2014.01.033 24518868

[B17] Carnevali L. Trombini M. Porta A. Montano N. De Boer S. F. Sgoifo A. (2013a). Vagal withdrawal and susceptibility to cardiac arrhythmias in rats with high trait aggressiveness. PLoS ONE 8:e68316 10.1371/journal.pone.0068316 23861886 PMC3701673

[B18] Carnevali L. Trombini M. Rossi S. Graiani G. Manghi M. Koolhaas J. M. (2013b). Structural and electrical myocardial remodeling in a rodent model of depression. Psychosom. Med. 75, 42–51 10.1097/PSY.0b013e318276cb0d 23257930

[B19] Cheng Z. Powley T. L. (2000). Nucleus ambiguus projections to cardiac ganglia of rat atria: an anterograde tracing study. J. Comp. Neurol. 424, 588–606 10.1002/1096-9861(20000904)424:4<588::AID-CNE3>3.0.CO;2-7 10931483

[B20] Cheng Z. Powley T. L. Schwaber J. S. Doyle F. J. 3rd. (1999). Projections of the dorsal motor nucleus of the vagus to cardiac ganglia of rat atria: an anterograde tracing study. J. Comp. Neurol. 410, 320–341 10414536

[B21] Chiladakis J. A. Pashalis A. Patsouras N. Manolis A. S. (2001). Autonomic patterns preceding and following accelerated idioventricular rhythm in acute myocardial infarction. Cardiology 96, 24–31 10.1159/000047382 11701937

[B22] Crimi E. Ignarro L. J. Cacciatore F. Napoli C. (2009). Mechanisms by which exercise training benefits patients with heart failure. Nat. Rev. Cardiol. 6, 292–300 10.1038/nrcardio.2009.8 19352333

[B23] Dutschmann M. Waki H. Manzke T. Simms A. E. Pickering A. E. Richter D. W. (2009). The potency of different serotonergic agonists in counteracting opioid evoked cardiorespiratory disturbances. Philos. Trans. R. Soc. Lond. B Biol. Sci. 364, 2611–2623 10.1098/rstb.2009.0076 19651661 PMC2865122

[B24] El-Sheikh M. Harger J. Whitson S. M. (2001). Exposure to interparental conflict and children's adjustment and physical health: the moderating role of vagal tone. Child Dev. 72, 1617–1636 10.1111/1467-8624.00369 11768136

[B25] Esler M. (1992). The autonomic nervous system and cardiac arrhythmias. Clin. Auton. Res. 2, 133–135 10.1007/BF01819669 1638108

[B26] Friedman B. H. Thayer J. F. (1998). Anxiety and autonomic flexibility: a cardiovascular approach. Biol. Psychol. 47, 243–263 10.1016/S0301-0511(97)00027-6 9564452

[B27] Fu Q. Levine B. D. (2013). Exercise and the autonomic nervous system. Handb. Clin. Neurol. 117, 147–160 10.1016/B978-0-444-53491-0.00013-4 24095123

[B28] Gorman J. M. Sloan R. P. (2000). Heart rate variability in depressive and anxiety disorders. Am. Heart J. 140, 77–83 10.1067/mhj.2000.109981 11011352

[B29] Grippo A. J. Beltz T. G. Weiss R. M. Johnson A. K. (2006). The effects of chronic fluoxetine treatment on chronic mild stress-induced cardiovascular changes and anhedonia. Biol. Psychiatry 59, 309–316 10.1016/j.biopsych.2005.07.010 16154542

[B30] Grippo A. J. Santos C. M. Johnson R. F. Beltz T. G. Martins J. B. Felder R. B. (2004). Increased susceptibility to ventricular arrhythmias in a rodent model of experimental depression. Am. J. Physiol. Heart Circ. Physiol. 286, H619–H626 10.1152/ajpheart.00450.2003 14715499

[B31] Hildreth C. M. Padley J. R. Pilowsky P. M. Goodchild A. K. (2008). Impaired serotonergic regulation of heart rate may underlie reduced baroreflex sensitivity in an animal model of depression. Am. J. Physiol. Heart Circ. Physiol. 294, H474–H480 10.1152/ajpheart.01009.2007 17993598

[B32] Huikuri H. V. Makikallio T. H. Airaksinen K. E. Seppanen T. Puukka P. Raiha I. J. (1998). Power-law relationship of heart rate variability as a predictor of mortality in the elderly. Circulation 97, 2031–2036 10.1161/01.CIR.97.20.2031 9610533

[B33] Huikuri H. V. Makikallio T. H. Peng C. K. Goldberger A. L. Hintze U. Moller M. (2000). Fractal correlation properties of R-R interval dynamics and mortality in patients with depressed left ventricular function after an acute myocardial infarction. Circulation 101, 47–53 10.1161/01.CIR.101.1.47 10618303

[B34] Iellamo F. Legramante J. M. Massaro M. Raimondi G. Galante A. (2000). Effects of a residential exercise training on baroreflex sensitivity and heart rate variability in patients with coronary artery disease: a randomized, controlled study. Circulation 102, 2588–2592 10.1161/01.CIR.102.21.2588 11085961

[B35] Jordan D. (2005). Vagal control of the heart: central serotonergic (5-HT) mechanisms. Exp. Physiol. 90, 175–181 10.1113/expphysiol.2004.029058 15604109

[B36] Jose A. D. Collison D. (1970). The normal range and determinants of the intrinsic heart rate in man. Cardiovasc. Res. 4, 160–167 10.1093/cvr/4.2.160 4192616

[B37] Kieran N. Ou X. M. Iyo A. H. (2010). Chronic social defeat downregulates the 5-HT1A receptor but not Freud-1 or NUDR in the rat prefrontal cortex. Neurosci. Lett. 469, 380–384 10.1016/j.neulet.2009.12.032 20026183 PMC2815082

[B38] Kleiger R. E. Stein P. K. Bosner M. S. Rottman J. N. (1992). Time domain measurements of heart rate variability. Cardiol. Clin. 10, 487–498 1504980

[B39] Kok B. E. Fredrickson B. L. (2010). Upward spirals of the heart: autonomic flexibility, as indexed by vagal tone, reciprocally and prospectively predicts positive emotions and social connectedness. Biol. Psychol. 85, 432–436 10.1016/j.biopsycho.2010.09.005 20851735 PMC3122270

[B40] Koolhaas J. M. Coppens C. M. De Boer S. F. Buwalda B. Meerlo P. Timmermans P. J. (2013). The resident-intruder paradigm: a standardized test for aggression, violence and social stress. J. Vis. Exp. 4:e4367 10.3791/4367 23852258 PMC3731199

[B41] Landgraf R. Wigger A. (2002). High vs low anxiety-related behavior rats: an animal model of extremes in trait anxiety. Behav. Genet. 32, 301–314 10.1023/A:1020258104318 12405513

[B42] La Rovere M. T. Bigger J. T. Jr. Marcus F. I. Mortara A. Schwartz P. J. (1998). Baroreflex sensitivity and heart-rate variability in prediction of total cardiac mortality after myocardial infarction. ATRAMI (Autonomic Tone and Reflexes After Myocardial Infarction) Investigators. Lancet 351, 478–484 10.1016/S0140-6736(97)11144-8 9482439

[B43] Leslie R. A. Reynolds D. J. Andrews P. L. Grahame-Smith D. G. Davis C. J. Harvey J. M. (1990). Evidence for presynaptic 5-hydroxytryptamine3 recognition sites on vagal afferent terminals in the brainstem of the ferret. Neuroscience 38, 667–673 10.1016/0306-4522(90)90060-H 2176720

[B44] Levy M. N. (1971). Sympathetic-parasympathetic interactions in the heart. Circ. Res. 29, 437–445 10.1161/01.RES.29.5.437 4330524

[B45] Levy M. N. Schwartz P. J. (1994). Vagal Control of the Heart: Experimental Basis and Clinical Implications. Armonk, NY: Futura Publishing Co

[B46] Lin Y. C. Horvath S. M. (1972). Autonomic nervous control of cardiac frequency in the exercise-trained rat. J. Appl. Physiol. 33, 796–799 4643860 10.1152/jappl.1972.33.6.796

[B47] Loewy A. D. (1990). Anatomy of the autonomic nervous system: an overview, in Central Regulation of the Autonomic Function, eds Loewy A. D. Spyer K. M. (New York, NY: Oxford University Press), 1–16

[B48] Lombardi F. Sandrone G. Mortara A. Torzillo D. La Rovere M. T. Signorini M. G. (1996). Linear and nonlinear dynamics of heart rate variability after acute myocardial infarction with normal and reduced left ventricular ejection fraction. Am. J. Cardiol. 77, 1283–1288 10.1016/S0002-9149(96)00193-2 8677867

[B49] Lucini D. Di Fede G. Parati G. Pagani M. (2005). Impact of chronic psychosocial stress on autonomic cardiovascular regulation in otherwise healthy subjects. Hypertension 46, 1201–1206 10.1161/01.HYP.0000185147.32385.4b 16203875

[B50] Makikallio T. H. Hoiber S. Kober L. Torp-Pedersen C. Peng C. K. Goldberger A. L. (1999). Fractal analysis of heart rate dynamics as a predictor of mortality in patients with depressed left ventricular function after acute myocardial infarction. TRACE Investigators. TRAndolapril Cardiac Evaluation. Am. J. Cardiol. 83, 836–839 10.1016/S0002-9149(98)01076-5 10190395

[B51] Makikallio T. H. Seppanen T. Airaksinen K. E. Koistinen J. Tulppo M. P. Peng C. K. (1997). Dynamic analysis of heart rate may predict subsequent ventricular tachycardia after myocardial infarction. Am. J. Cardiol. 80, 779–783 10.1016/S0002-9149(97)00516-X 9315590

[B52] Mann J. J. Stoff D. M. (1997). A synthesis of current findings regarding neurobiological correlates and treatment of suicidal behavior. Ann. N.Y. Acad. Sci. 836, 352–363 10.1111/j.1749-6632.1997.tb52370.x 9616809

[B53] Medeiros A. Oliveira E. M. Gianolla R. Casarini D. E. Negrao C. E. Brum P. C. (2004). Swimming training increases cardiac vagal activity and induces cardiac hypertrophy in rats. Braz. J. Med. Biol. Res. 37, 1909–1917 10.1590/S0100-879X2004001200018 15558199

[B54] Mendelowitz D. (1996). Firing properties of identified parasympathetic cardiac neurons in nucleus ambiguus. Am. J. Physiol. 271, H2609–H2614 8997322 10.1152/ajpheart.1996.271.6.H2609

[B55] Mezzacappa E. S. Kelsey R. M. Katkin E. S. Sloan R. P. (2001). Vagal rebound and recovery from psychological stress. Psychosom. Med. 63, 650–657 11485119 10.1097/00006842-200107000-00018

[B56] Miczek K. A. (1979). A new test for aggression in rats without aversive stimulation: differential effects of d-amphetamine and cocaine. Psychopharmacology 60, 253–259 10.1007/BF00426664 108702

[B57] Mueller P. J. Hasser E. M. (2006). Putative role of the NTS in alterations in neural control of the circulation following exercise training in rats. Am. J. Physiol. Regul. Integr. Comp. Physiol. 290, R383–R392 10.1152/ajpregu.00455.2005 16179489

[B58] Nalivaiko E. (2006). 5-HT(1A) receptors in stress-induced cardiac changes: a possible link between mental and cardiac disorders. Clin. Exp. Pharmacol. Physiol. 33, 1259–1264 10.1111/j.1440-1681.2006.04521.x 17184512

[B59] Nalivaiko E. Sgoifo A. (2009). Central 5-HT receptors in cardiovascular control during stress. Neurosci. Biobehav. Rev. 33, 95–106 10.1016/j.neubiorev.2008.05.026 18573276

[B60] Neto O. B. Abate D. T. Junior M. M. Mota G. R. Orsatti F. L. Silva R. C. (2013). Exercise training improves cardiovascular autonomic activity and attenuates renal damage in spontaneously hypertensive rats. J. Sports Sci. Med. 12, 52–59 24149725 PMC3761757

[B61] Netzer F. Bernard J. F. Verberne A. J. Hamon M. Camus F. Benoliel J. J. (2011). Brain circuits mediating baroreflex bradycardia inhibition in rats: an anatomical and functional link between the cuneiform nucleus and the periaqueductal grey. J. Physiol. 589, 2079–2091 10.1113/jphysiol.2010.203737 21486808 PMC3090605

[B62] Ngampramuan S. Baumert M. Beig M. I. Kotchabhakdi N. Nalivaiko E. (2008). Activation of 5-HT(1A) receptors attenuates tachycardia induced by restraint stress in rats. Am. J. Physiol. Regul. Integr. Comp. Physiol. 294, R132–R141 10.1152/ajpregu.00464.2007 17959705

[B63] Nolan R. P. Jong P. Barry-Bianchi S. M. Tanaka T. H. Floras J. S. (2008). Effects of drug, biobehavioral and exercise therapies on heart rate variability in coronary artery disease: a systematic review. Eur. J. Cardiovasc. Prev. Rehabil. 15, 386–396 10.1097/HJR.0b013e3283030a97 18677161

[B64] Parsey R. V. Oquendo M. A. Ogden R. T. Olvet D. M. Simpson N. Huang Y. Y. (2006). Altered serotonin 1A binding in major depression: a [carbonyl-C-11]WAY100635 positron emission tomography study. Biol. Psychiatry 59, 106–113 10.1016/j.biopsych.2005.06.016 16154547

[B65] Porges S. W. (1995a). Cardiac vagal tone: a physiological index of stress. Neurosci. Biobehav. Rev. 19, 225–233 10.1016/0149-7634(94)00066-A 7630578

[B66] Porges S. W. (1995b). Orienting in a defensive world: mammalian modifications of our evolutionary heritage. A Polyvagal Theory. Psychophysiology 32, 301–318 10.1111/j.1469-8986.1995.tb01213.x 7652107

[B67] Porges S. W. (2007). The polyvagal perspective. Biol. Psychol. 74, 116–143 10.1016/j.biopsycho.2006.06.009 17049418 PMC1868418

[B68] Rennie K. L. Hemingway H. Kumari M. Brunner E. Malik M. Marmot M. (2003). Effects of moderate and vigorous physical activity on heart rate variability in a British study of civil servants. Am. J. Epidemiol. 158, 135–143 10.1093/aje/kwg120 12851226

[B69] Rentero N. Cividjian A. Trevaks D. Pequignot J. M. Quintin L. McAllen R. M. (2002). Activity patterns of cardiac vagal motoneurons in rat nucleus ambiguus. Am. J. Physiol. Regul. Integr. Comp. Physiol. 283, R1327–R1334 10.1152/ajpregu.00271.2002 12388471

[B70] Reyes Del Paso G. A. Langewitz W. Mulder L. J. Van Roon A. Duschek S. (2013). The utility of low frequency heart rate variability as an index of sympathetic cardiac tone: a review with emphasis on a reanalysis of previous studies. Psychophysiology 50, 477–487 10.1111/psyp.12027 23445494

[B71] Rosenwinkel E. T. Bloomfield D. M. Arwady M. A. Goldsmith R. L. (2001). Exercise and autonomic function in health and cardiovascular disease. Cardiol. Clin. 19, 369–387 10.1016/S0733-8651(05)70223-X 11570111

[B72] Rossi B. R. Mazer D. Silveira L. C. Jacinto C. P. Di Sacco T. H. Blanco J. H. (2009). Physical exercise attenuates the cardiac autonomic deficit induced by nitric oxide synthesis blockade. Arq. Bras. Cardiol. 92, 31–38 10.1590/S0066-782X2009000100006 19219262

[B73] Rozanski A. Blumenthal J. A. Kaplan J. (1999). Impact of psychological factors on the pathogenesis of cardiovascular disease and implications for therapy. Circulation 99, 2192–2217 10.1161/01.CIR.99.16.2192 10217662

[B74] Sabbah H. N. Ilsar I. Zaretsky A. Rastogi S. Wang M. Gupta R. C. (2011). Vagus nerve stimulation in experimental heart failure. Heart Fail. Rev. 16, 171–178 10.1007/s10741-010-9209-z 21128115 PMC3784341

[B75] Sant'Ana J. E. Pereira M. G. Dias Da Silva V. J. Dambros C. Costa-Neto C. M. Souza H. C. (2011). Effect of the duration of daily aerobic physical training on cardiac autonomic adaptations. Auton. Neurosci. 159, 32–37 10.1016/j.autneu.2010.07.006 20708981

[B76] Schwartz P. J. Vanoli E. Stramba-Badiale M. De Ferrari G. M. Billman G. E. Foreman R. D. (1988). Autonomic mechanisms and sudden death. New insights from analysis of baroreceptor reflexes in conscious dogs with and without a myocardial infarction. Circulation 78, 969–979 10.1161/01.CIR.78.4.969 3168199

[B77] Sevoz-Couche C. Brouillard C. Camus F. Laude D. De Boer S. F. Becker C. (2013). Involvement of the dorsomedial hypothalamus and the nucleus tractus solitarii in chronic cardiovascular changes associated with anxiety in rats. J. Physiol. 591, 1871–1887 10.1113/jphysiol.2012.247791 23297312 PMC3624857

[B78] Sgoifo A. Carnevali L. Grippo A. J. (2014). The socially stressed heart. Insights from studies in rodents. Neurosci. Biobehav. Rev. 39C, 51–60 10.1016/j.neubiorev.2013.12.005 24373860

[B79] Sgoifo A. De Boer S. F. Buwalda B. Korte-Bouws G. Tuma J. Bohus B. (1998). Vulnerability to arrhythmias during social stress in rats with different sympathovagal balance. Am. J. Physiol. 275, H460–H466 9683433 10.1152/ajpheart.1998.275.2.H460

[B80] Sgoifo A. Stilli D. Medici D. Gallo P. Aimi B. Musso E. (1996). Electrode positioning for reliable telemetry ECG recordings during social stress in unrestrained rats. Physiol. Behav. 60, 1397–1401 10.1016/S0031-9384(96)00228-4 8946481

[B81] Sloan R. P. Shapiro P. A. Bigger J. T. Jr. Bagiella E. Steinman R. C. Gorman J. M. (1994). Cardiac autonomic control and hostility in healthy subjects. Am. J. Cardiol. 74, 298–300 10.1016/0002-9149(94)90382-4 8037145

[B82] Smeets T. Giesbrecht T. Raymaekers L. Shaw J. Merckelbach H. (2010). Autobiographical integration of trauma memories and repressive coping predict post-traumatic stress symptoms in undergraduate students. Clin. Psychol. Psychother. 17, 211–218 10.1002/cpp.644 19701880

[B83] Smith M. L. Hudson D. L. Graitzer H. M. Raven P. B. (1989). Exercise training bradycardia: the role of autonomic balance. Med. Sci. Sports Exerc. 21, 40–44 10.1249/00005768-198902000-00008 2927300

[B84] Soares-Miranda L. Sandercock G. Valente H. Vale S. Santos R. Mota J. (2009). Vigorous physical activity and vagal modulation in young adults. Eur. J. Cardiovasc. Prev. Rehabil. 16, 705–711 10.1097/HJR.0b013e3283316cd1 19738471

[B85] Souza G. G. Magalhaes L. N. Cruz T. A. Mendonca-De-Souza A. C. Duarte A. F. Fischer N. L. (2013). Resting vagal control and resilience as predictors of cardiovascular allostasis in peacekeepers. Stress 16, 377–383 10.3109/10253890.2013.767326 23327672

[B86] Souza H. C. De Araujo J. E. Martins-Pinge M. C. Cozza I. C. Martins-Dias D. P. (2009). Nitric oxide synthesis blockade reduced the baroreflex sensitivity in trained rats. Auton. Neurosci. 150, 38–44 10.1016/j.autneu.2009.04.007 19443278

[B87] Stein P. K. Bosner M. S. Kleiger R. E. Conger B. M. (1994). Heart rate variability: a measure of cardiac autonomic tone. Am. Heart J. 127, 1376–1381 10.1016/0002-8703(94)90059-0 8172068

[B88] Task Force of the European Society of Cardiology and the North American Society of Pacing and Electrophysiology. (1996). Heart rate variability: standards of measurement, physiological interpretation and clinical use. Circ. Res. 93, 1043–1065 10.1161/01.CIR.93.5.1043 8598068

[B89] Trombini M. Hulshof H. J. Graiani G. Carnevali L. Meerlo P. Quaini F. (2012). Early maternal separation has mild effects on cardiac autonomic balance and heart structure in adult male rats. Stress 15, 457–470 10.3109/10253890.2011.639414 22085295

[B90] Tsai M. W. Chie W. C. Kuo T. B. Chen M. F. Liu J. P. Chen T. T. (2006). Effects of exercise training on heart rate variability after coronary angioplasty. Phys. Ther. 86, 626–635 16649887

[B91] Volders P. G. (2010). Novel insights into the role of the sympathetic nervous system in cardiac arrhythmogenesis. Heart Rhythm 7, 1900–1906 10.1016/j.hrthm.2010.06.003 20570754

[B92] Wood S. K. McFadden K. V. Grigoriadis D. Bhatnagar S. Valentino R. J. (2012). Depressive and cardiovascular disease comorbidity in a rat model of social stress: a putative role for corticotropin-releasing factor. Psychopharmacology 222, 325–336 10.1007/s00213-012-2648-6 22322324 PMC3613282

